# Three-dimensional analysis of the root canal preparation with Reciproc Blue®, WaveOne Gold® and XP EndoShaper®: a new method in vivo

**DOI:** 10.1186/s12903-021-01450-1

**Published:** 2021-02-25

**Authors:** Javier Caviedes-Bucheli, Nestor Rios-Osorio, Diana Usme, Cristian Jimenez, Adriana Pinzon, Jorge Rincón, María M. Azuero-Holguin, Alvaro Zubizarreta-Macho, Jose F. Gomez-Sosa, Hugo R. Munoz

**Affiliations:** 1 Centro de Investigaciones Odontologicas, Pontificia Universidad Javeriana, Bogota, Colombia; 2 Postgraduate Endodontics Department, Institucion Universitaria Colegios de Colombia, Bogota, Colombia; 3 Private Practice, Bogota, Colombia; 4 Department of Endodontics, Faculty of Health Sciences, Alfonso X El Sabio University, Madrid, Spain; 5 Postgraduate Endodontics Department, Universidad Central de Venezuela, Caracas, Venezuela; 6 Endodontics Department, Universidad de San Carlos, Guatemala, Guatemala

**Keywords:** Cone-beam computed tomography, 3D reconstruction, Root canal preparation, Single-file, Reciproc Blue, WaveOne Gold, XP-EndoShaper

## Abstract

**Background:**

The purpose of this study was to evaluate the changes in canal volume after root canal preparation in vivo with 3 different single-file techniques (Reciproc-Blue®, WaveOne-Gold® and XP-EndoShaper®), with a new method using CBCT and 3D reconstruction.

**Methods:**

In this prospective study, thirty human lower premolars from healthy patients were used, in which extraction was indicated for orthodontic reasons. All the teeth used were caries- and restoration-free with complete root development, without signs of periodontal disease or traumatic occlusion, and with only one straight canal (up to 25º curvature). Teeth were randomly divided into three different groups: Reciproc-Blue, WaveOne-Gold and XP-EndoShaper. CBCT scans before root canal preparation were used to create a 3D reconstruction with RHINOCEROS 5.0 software to assess the initial canal volume, and then compared with 3D reconstructions after canal preparation to measure the increase in canal volume. Student’s t test for paired data were used to determine statistically significant differences between the before and after canal volumes. Anova test was used to determine statistically significant differences in the percentage of canal volume increase between the groups and Tukey's post-hoc test were used to paired comparison.

**Results:**

Reciproc-Blue showed the higher increase in canal volume, followed by WaveOne-Gold and XP-EndoShaper (p = 0.003). XP-EndoShaper did not show a statistically significant increase in canal volume after root canal preparation (p = 0.06).

**Conclusion:**

With this model, Reciproc-Blue showed higher increase in root canal volume, followed by WaveOne-Gold, while XP-EndoShaper did not significantly increase root canal volume during preparation.

## Background

Optimal endodontic preparation aims to preserve the original morphology of root canals, respecting the size and spatial position of the apical foramen [1]. Operative procedural errors, such as over instrumentation and poor instrumentation, could lead to alterations in the canal volume [2–4].

The internal cross-section anatomy of root canals has different shapes and sizes, being oval shapes the most common at the cervical and middle thirds, while rounded shapes are more common at the apical third. These variations in the internal anatomy of the canal makes cleaning and disinfecting difficult [5, 6].

Nickel–Titanium (NiTi) instrument appearance during the 1990s intended to avoid several shortcomings of stainless-steel hand file instrumentation techniques seeking to maintain the canal´s original shape after root canal preparation [7]. However, most of the NITI rotary systems have been designed to work within the central portion of the canal; generating a round-shaped preparation, leaving parts of the canal walls untouched [8]. Therefore, the shaping ability of NITI rotary files in oval-shaped canals is still a main endodontic concern, since unprepared root canal areas harbor necrotic endodontic tissue or microbial biofilms, risking the treatment outcome [9].

Manufacturers claim that novel file designs with modified geometries and expandable profile, asymmetrical rotary motion, the reciprocating movement, and advancements in the thermomechanical treatment of the NITI alloys, such as gold (Waveone Gold®), blue (Reciproc Blue®), and maxwire (XP Endoshaper®) heat-treated files, improve the adaptation of endodontic files to the root canal anatomy while maintaining its original shape [9–11].

Assessment of the ability of NiTi files to prepare the root canal in terms of canal volume of prepared and unprepared areas and changes in canal morphology can be accurately examined using Microcomputed tomography (Micro CT) or cone-beam computed tomography (CBCT) [9]. Micro CT is a high-resolution imaging device, which produces 3D images of a biological structure. Micro CT is currently considered the gold standard for the evaluation of root canal microarchitecture, as morphological measurements by micro-CT reconstruction highly correlate with histo-morphometric results in a reproducible way. However, the impossibility to use micro-CT to scan teeth in vivo limits its usage [9].

CBCT is a modification of the former computed tomography (CT) systems. The CBCT is a reproducible method that captures data directly from the patient by using a cone-shaped X-ray beam. The data are then used to reconstruct a three-dimensional (3D) image of soft and mineralized tissues without causing any harm to tooth anatomy, obtaining high resolution images. CBCT has several advantages over micro-CT, such as a lower radiation dose, shorter scanning time and reduced cost [12].

Many studies have been conducted to evaluate root canal preparation with different files systems [1, 3–5]. All of these studies used ex vivo models, such as simulated canals [1, 4] and extracted teeth [3, 5] to observe the variation created within the root canal. The main tool used in most of these studies was the Micro CT which has been considered the gold standard in 3D reconstruction to evaluate root canal preparation [3, 5]. However, although this technology is inapplicable in patients, the results have been considered valid and applicable to clinical practice.

This in vivo study is based on the use of a reproducible method, the cone-beam computed tomography (CBCT), which provides three-dimensional high resolution and precise digital images with significant reduction of exposure time and low radiation dose. It allows to evaluate different anatomical aspects in relation to root canal preparation, eliminating the superposition of images. Recent in vivo studies have reported a greater sensitivity and specificity of these diagnostic images, and they can be used for the analysis of the radicular anatomy as well, with better application in clinical practice [13, 14]. Moreover, these images can be used to generate 3D reconstruction images with different design software.

The model presented also used the Rhinoceros 5.0 3D program, which is a software tool for drawing and modelling in 3D used in naval engineering. It allows to reconstruct curves and surfaces, creating polygonal meshes of real objects, and therefore is capable to reconstruct pre- and post-instrumentation anatomies of the root canal [15, 16]. Rhinoceros software allows to create precise mathematical representation of freeform surfaces and curves in computer graphics, which could be very useful for 3D reconstruction of teeth and root canals. This software has been successfully tested in medical [17, 18] and dental applications [19–21].

Therefore, the purpose of this study is to evaluate in vivo the changes in canal volume after root canal preparation with 3 different single-file techniques (Reciproc-Blue®, WaveOne-Gold® and XP-EndoShaper®), with a new method using CBCT and 3D reconstructions.

## Methods

A prospective in vivo experimental study was performed following the recommendations of the 8430 resolution of the Colombian Ministry of Health regarding ethical issues in research involving humans or their tissues. It was approved by the bioethics committee of the University Colegios de Colombia (RN27/02/22/2017). Written informed consent was obtained from each patient participating in the study (18–30 years old, healthy, not medicated, and non-smoking human donors). This study was also made following the CBCT use guidelines in clinical practice [22].

### Inclusion and exclusion criteria

Patients selected were going through orthodontic treatment with indication of double CBCT scans, one for the diagnosis of dento-maxillofacial anomalies and the other for the control of orthodontic treatment [23]. Thirty lower premolars were selected from these patients, in which extraction was indicated for orthodontic reasons. All the teeth used were caries- and restoration-free with complete root development determined both clinically and radiographically, without signs of periodontal disease or traumatic occlusion. Teeth had only one straight canal (canal curvatures over 25º were not included). Each premolar was randomly assigned for one of the experimental groups, consisting of 10 premolars each: (a) Reciproc Blue (VDW, Munich, Germany); (b) WaveOne Gold (Dentsply/Maillefer, Ballaigues, Switzerland); and (c) XP EndoShaper (FKG/Dentaire, La-Chaux-de-Fonds, Switzerland). The sample size was estimated based on the behaviour of canal volume change variables on in vitro studies and confirmed with the TAMAMU 1.1® program (Tokyo, Japan).

### Clinical and radiographical procedures

The initial CBCT was taken with the Carestream Dental CS 8100 3D (CARECAPITAL ADVISORS LIMITED / Rochester, New York, United States), with a 100 kVp voltage and 3–8 mGy / cm2 current, using a 75 µm minimum isometric cubic voxel size; and a gray value range of 14 bits using the CS 3D software and sensor CMOS 4 T. The scan time was approximately 10 s for each patient. Images of the selected premolars were analyzed with the Nobel-clinician software (Nobel Biocare Inc, USA).

All patients underwent prophylaxis with hydrogen peroxide and prophylactic brush, then they were anaesthetized with an inferior alveolar nerve block technique using 1.8 mL of 4% prilocaine without vasoconstrictor. Rubber dam isolation was placed and the cavity access was performed with a Zekrya bur. Canal patency was confirmed with a #10 K file (Dentsply/Maillefer, Ballaigues, Switzerland), working length was established (at -0.5 mm from apical foramen) with the Root ZX apex locator (J Morita, Japan) and verified with a periapical radiography. The root canal samples were prepared with the correspondent technique for each group following the manufacturer’s instructions, using a VDW Silver Reciproc endodontic motor (VDW, Munich, Germany) as follows:

#### Reciproc Blue group

The root canal was prepared using one new Reciproc Blue size 25/0.08 file (VDW, Munich, Germany) activated in a VDW Silver Reciproc motor (VDW, Munich, Germany) at the RECIPROC ALL setting, following the manufacturer’s recommendations. The file was used with short up and down motion with slight apical pressure in three cycles, one to prepare each third of the canal (cervical, middle and apical). After each cycle, the file was cleaned with wet gauze to remove dentine debris, and the canal was irrigated with 3 mL of 5.25% sodium hypochlorite (NaOCl) using a Monoject syringe with a 30-gauge needle placed 2 mm short of working length to complete a total of 9 mL of NaOCl for each canal. Effective working time of the file inside the canal did not exceed 1 min.

#### WaveOne Gold group

The root canal was prepared using one new WaveOne Gold primary (size 25/0.07) file (Dentsply/Maillefer, Ballaigues, Switzerland) activated in a VDW Silver Reciproc motor (VDW, Munich, Germany), at the WAVEONE ALL setting, following the manufacturer’s recommendations. Irrigation volume and effective working time of the file inside the canal were the same as described for the Reciproc Blue group.

#### XP EndoShaper group

The root canal was prepared using one new XP EndoShaper size 30/0.01 (FKG/Dentaire, La-Chaux-de-Fonds, Switzerland) activated in a VDW silver motor (VDW, Munich, Germany) strictly following the manufacturer’s recommendations. Irrigation volume and effective working time of the file inside the canal were the same as described for the Reciproc Blue and WaveOne Gold groups.

A second tomographic analysis was performed with CBCT, taking advantage of orthodontic control for dento-maxillofacial anomalies presented in the selected patients that needed to be followed up. Following the same steps as the initial CBCT, a digital file in 3dm format was obtained with the reconstruction of the sample after root canal preparation to carry out the superposition of preparation images before and after in order to evaluate the variables proposed in the study.

### 3D reconstruction process

**Fig. 1:**
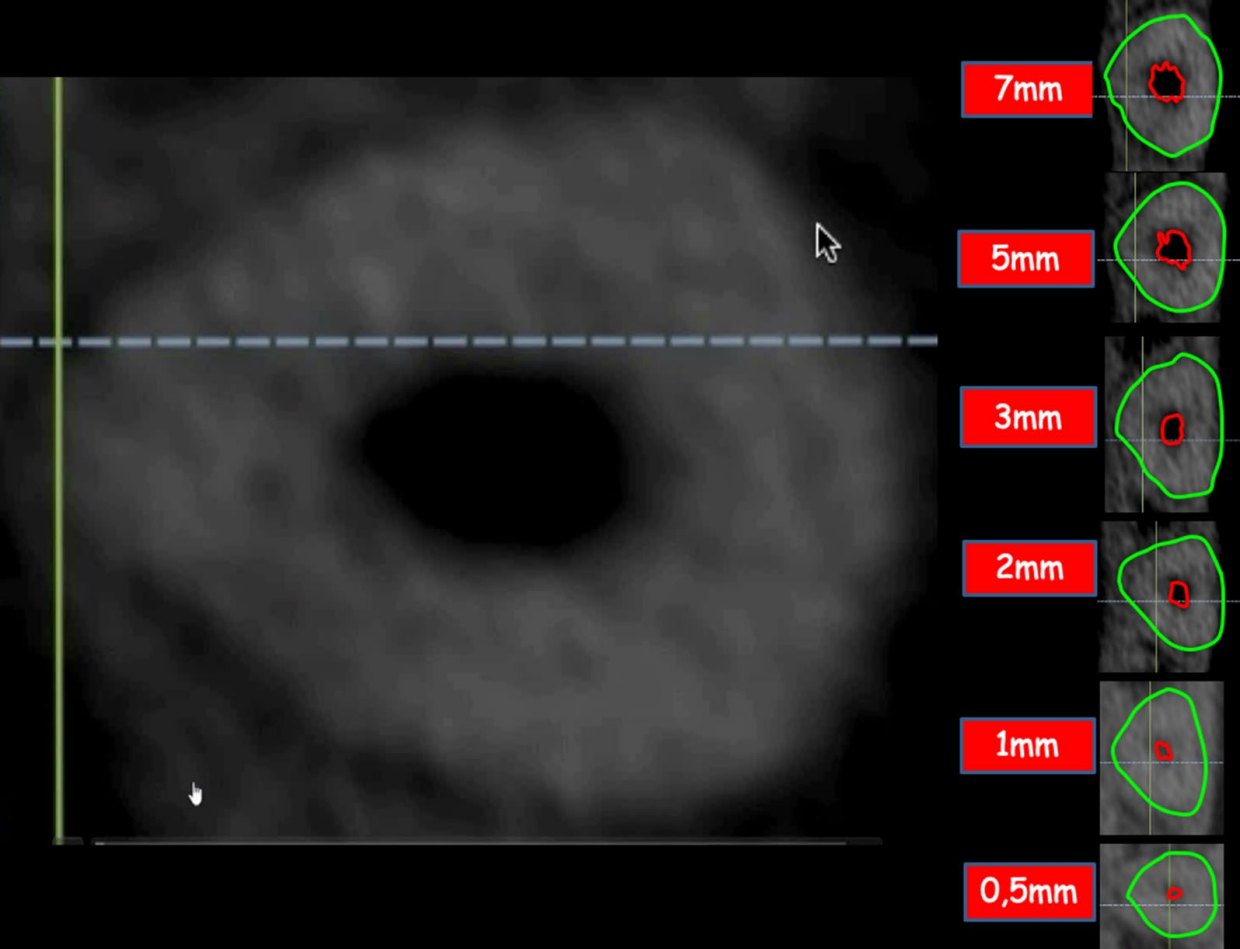
CBCT axial plane slices made at 0.5 mm, 1 mm, 2 mm, 3 mm, 4 mm, 5 mm, 6 mm and 7 mm, where measures of the root canal and the root were taken for three-dimensional reconstruction. Snapshot images of the different sections were imported with the RHINOCEROS 5.0 3D software to draw the canal and the root

**Fig. 2:**
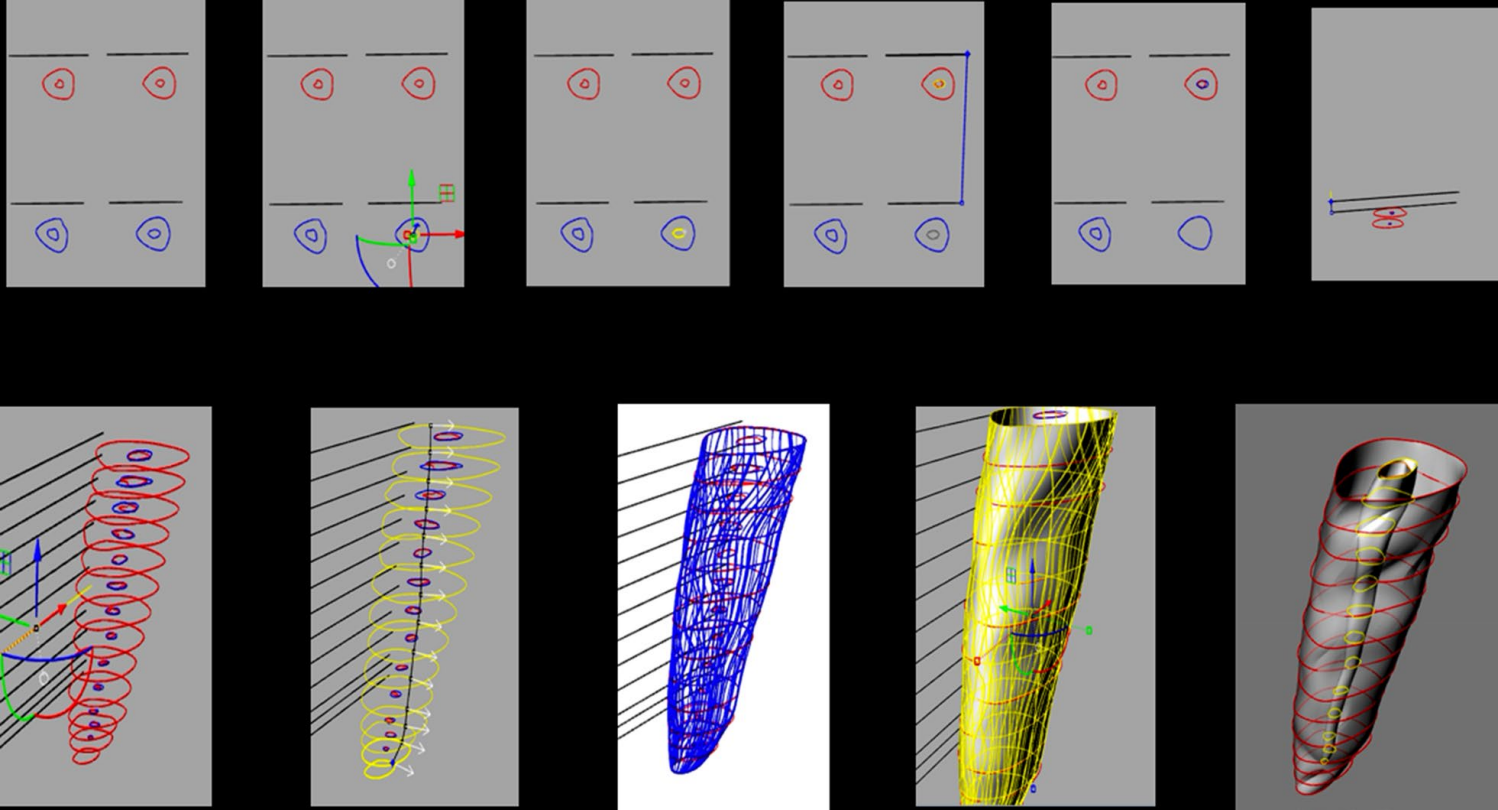
3D-reconstruction procedure step by step

**Fig. 3:**
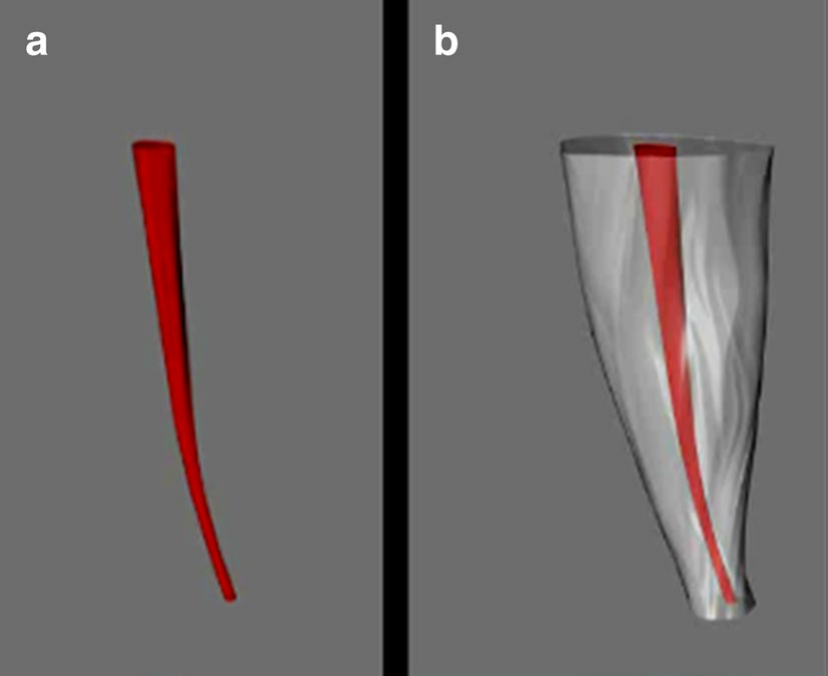
Reconstruction of the sample previous to root canal preparation. **a** 3D root canal reconstruction; **b** 3D root reconstruction with unprepared canal

Sixty CBCT scans of the teeth were obtained from before and after root canal preparations. At the axial plane, slices were made at 0.5 mm, 1 mm, 2 mm, 3 mm, 4 mm, 5 mm, 6 mm and 7 mm, and measures of the root canal and the root were taken from vestibular to palatal and from mesial to distal for three-dimensional reconstruction. Snapshot images of the different sections were imported with the Rhinoceros 3D 5.0 software (Robert McNeel & Associates, Washington, USA) to draw the canal and the root using the poly-line command (Figs. 1, 2). A digital file in 3dm format was obtained with the reconstruction of the teeth previous to root canal preparation [13, 15] (Fig. 3).

**Fig. 4:**
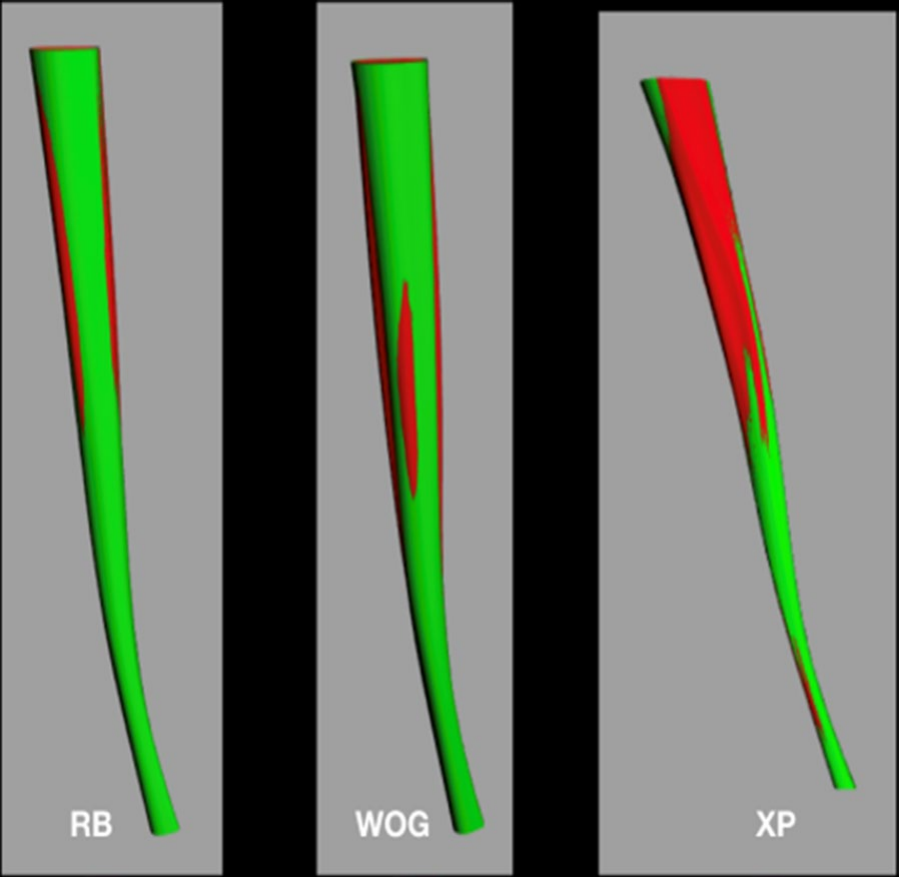
Superimposition of before and after preparation images with different systems. RB = Reciproc Blue; WOG = WaveOne Gold; XP = XP EndoShaper

The tomography was framed with the poly-line command, to be used later as a reference point. These slices with the frame were exported to the Rhinoceros software (Robert McNeel & Associates, Washington, USA) one by one, both of the root and the canal. Also, a reference line was drawn connecting the point of intersection of the aforementioned lines in the root and the canal, to make sure that the position of the canal inside the root was not altered in the previous steps (Fig. 4).

**Fig. 5:**
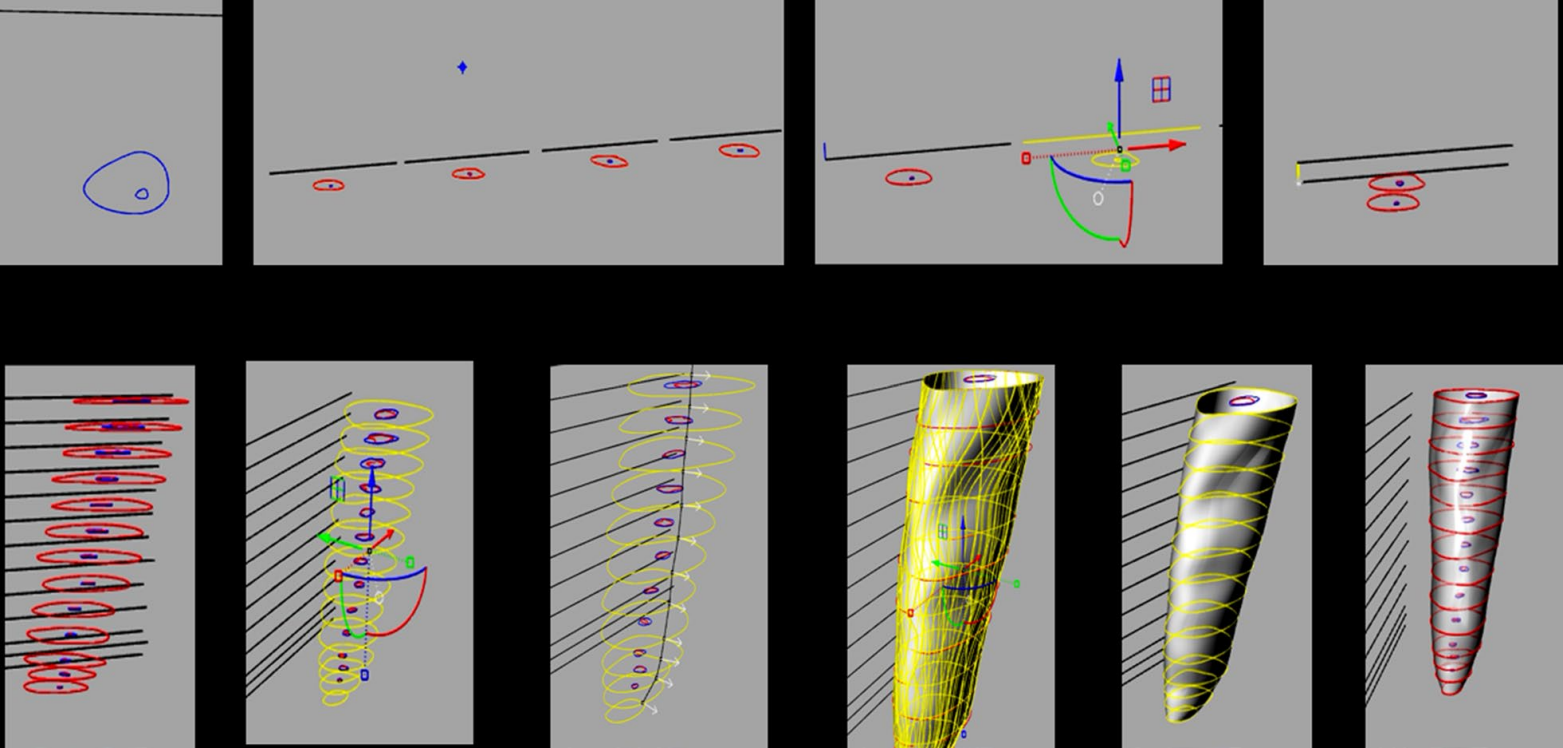
Procedure of superimposition step by step

With the guide lines and the table of measurements, each root and each canal was given its corresponding measure in μm with the scale 1D command, enlarging or reducing the drawing according to the measurements table. All the reference lines used were removed to clean the drawing. After all the slices were scaled, it was proceeded to join each pre-operative slice to its corresponding original millimeter. This was done with the move command using both the frame of the tomography, that was preserved at the beginning, and the root itself as reference points (Fig. 5).

**Fig. 6:**
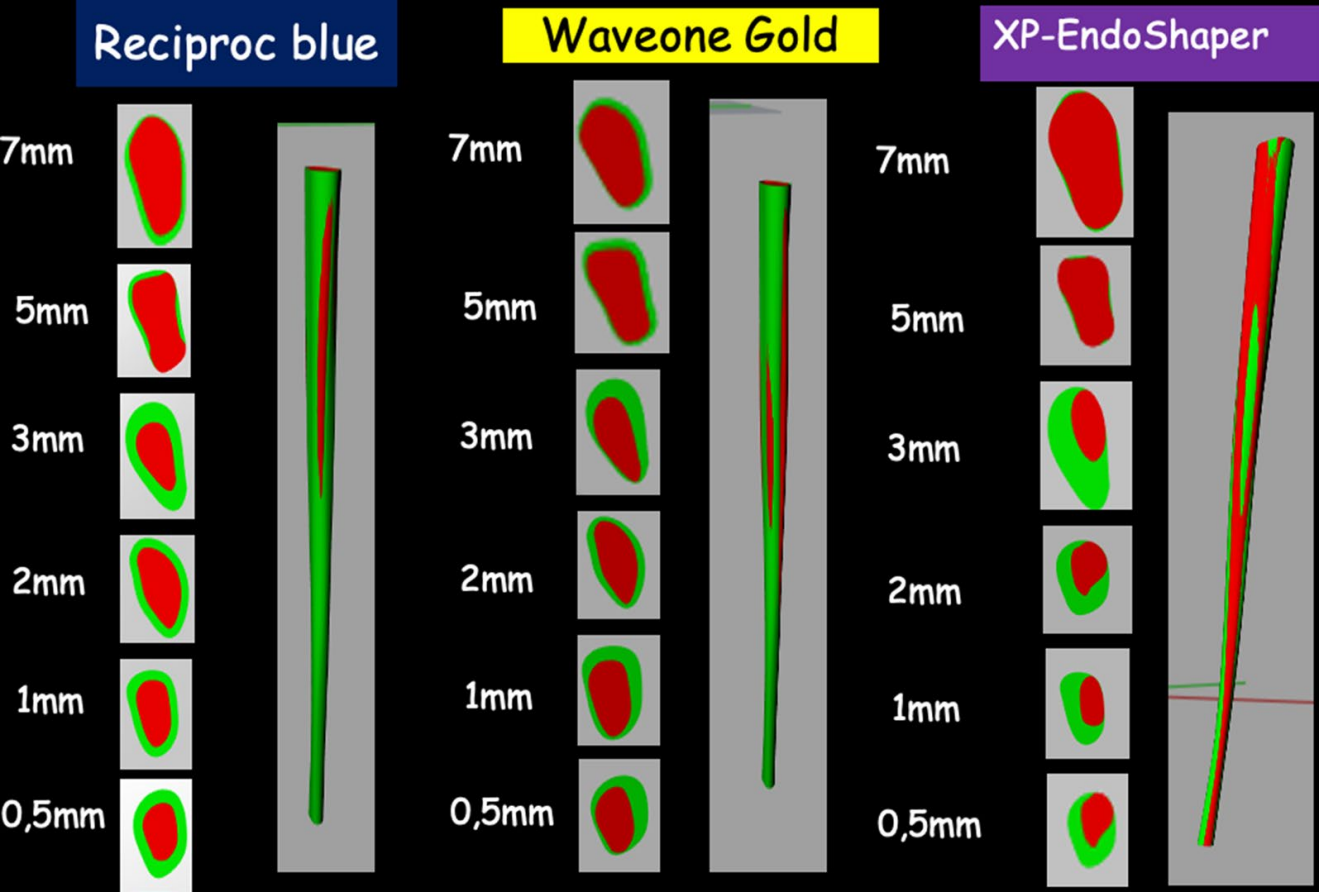
Examples of final 3D reconstructions with details such as the canal colors of teeth prepared with different systems. RB = Reciproc Blue; WOG = WaveOne Gold; XP = XP EndoShaper

The above procedure reduces the number of slices to 6 and root canals were located both before and after the endodontic preparation. The next step was to place the three dimensionally slices on top of each other at the corresponding heights with the move command, giving a diagram of millimeter-by-millimeter heights of the root and the canal. When having the slices in this position, a complex surface was created between all slices for each element with the loft command and the result was a surface for the root, one for the original canal and one for the prepared canal (Fig. 6).

**Fig. 7:**
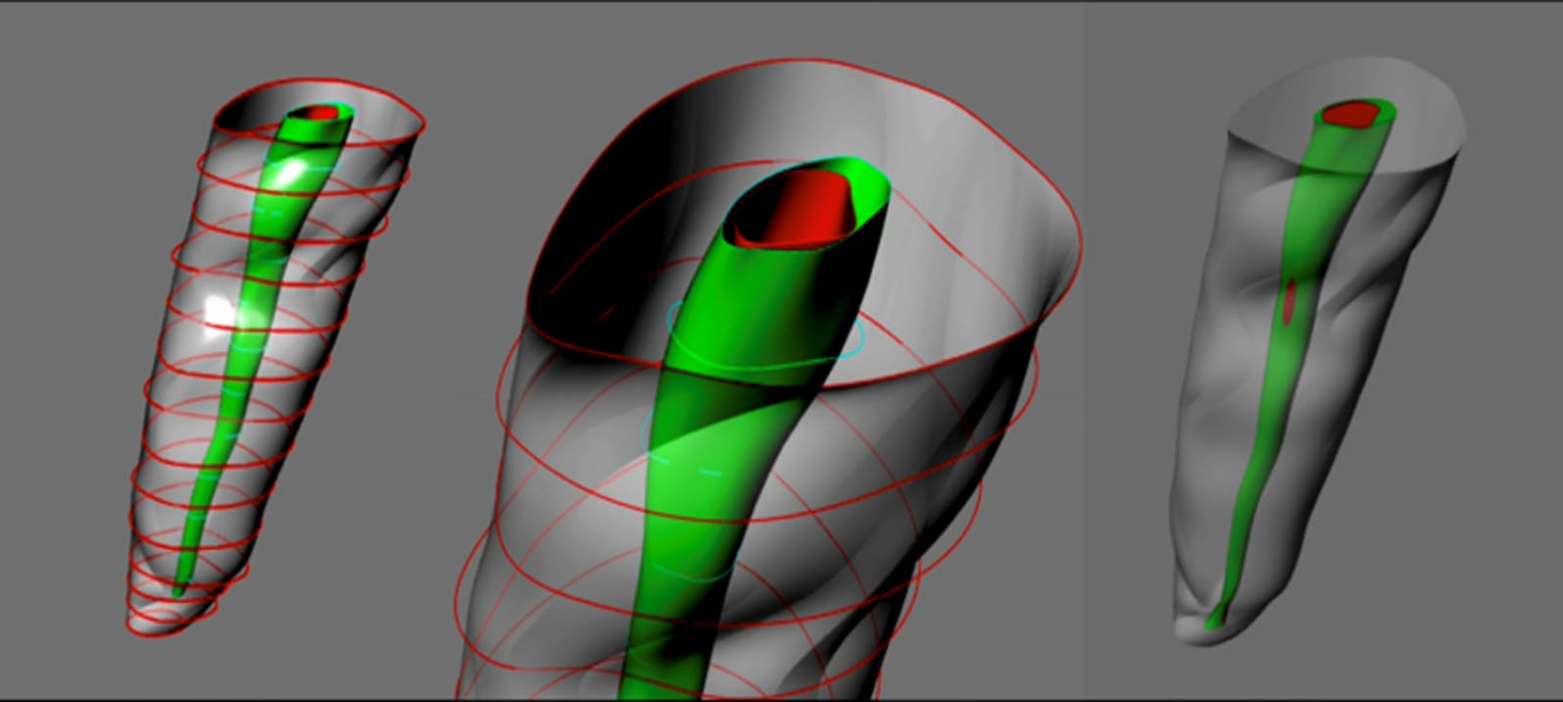
Three-dimensional drawing of root contour and root canal before (red) and after (green) preparation with the Rhinoceros 5.0 3D software using exact measures taken from the CBCT slices

**Fig. 8:**
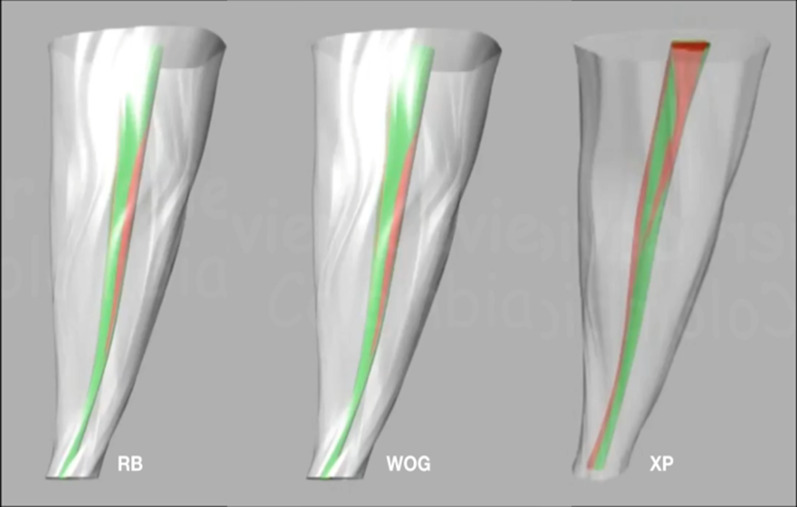
Examples of final 3D reconstructions with details such as the canal colors and the transparency of the roots with prepared canal. RB = Reciproc Blue; WOG = WaveOne Gold; XP = XP EndoShaper

Three surfaces remain that are then covered with the plane and split commands to generate a solid form. Finally, the edge of the canals was created following an hourglass shape with the loft command, this was covered with the commands mentioned above and details, such as the colors and the transparency of the root, were added with the material editor command (Figs. 7, 8).

### Outcome variables

The total volume of the root canal was measured before and after root canal preparation by using the volume command in the Rhinoceros 5.0 software. This function gives the volume result of a solid in mm^3^. Percentage of volume increase was also calculated to compare the volume increase for each group.

### Statistical analysis

Student t test analysis for paired data were used to determine statistically significant differences between the before and after canal volumes. Anova test was used to determine statistically significant differences in the percentage of canal volume increase between the experimental groups. Finally, Tukey’s HSD post hoc tests were used for paired comparisons between groups.

## Results

All experimental teeth could be successfully 3D digitalized through Rhino software. These images clearly showed the cutting ability of the three files systems tested, by the superimposition of the canal reconstruction before and after the canal preparation.

Table 1 shows the comparison between the initial volume of the canal prior to the instrumentation procedure and the final volume after preparation. It can be noticed that the original canals volume of the 3 groups were similar, as there was no statistically significant difference between the volume of the pre-instrumented canals that were assigned to each group (p = 0.87). Student t test analysis for paired data showed statistically significant canal volume increases in the Reciproc Blue and WaveOne Gold groups (p < 0.001). The XP EndoShaper group did not show significant differences between the canal volume before and after the instrumentation (p = 0.06).

**Table 1:** Canal Volume in mm^3^ before and after preparation with three different systems

	N	Canal volume before preparation*	Canal volume after preparation**	Paired T-student
Reciproc Blue***	10	8.158 ± 5.16	14.692 ± 6.37	p < 0.001
WaveOne Gold****	10	7.527 ± 3.47	10.933 ± 2.65	p < 0.001
XP EndoShaper****	10	8.638 ± 4.53	9.873 ± 4.74	p = 0.06

^*^ Anova p = 0.87

^**^Anova p = 0.03

^***^Tukey post-hoc test showed significant difference between Reciproc Blue and the other two systems (p > 0.05)

^****^No significant differences were observed between WaveOne Gold and XP EndoShaper (p > 0.05)

Additionally, the percentage of canal volume increase was calculated after preparation with each system. Table 2 shows that Reciproc Blue produces the largest canal volume increase with a mean 110.34% ± 72.4%, followed by WaveOne Gold with a mean volume increase of 81.60% ± 63.6%. XP EndoShaper produced the least canal volume increase with a mean of 17.55% ± 9.6%. ANOVA test showed statistically significant differences between groups (p = 0.003). Tukey’s test post-hoc comparisons revealed statistically significant differences between XP EndoShaper and the two other groups (p < 0.05). No significant differences were observed between Reciproc Blue and WaveOne Gold groups (p = 0.508).

**Table 2:** Percentage of canal volume increase after preparation with three different systems

	N	Mean*	Standard deviation	Minimum	Maximum
Reciproc Blue**	10	110.34	72.47	51.12	238.20
WaveOne Gold**	10	81.61	63.62	22.22	169.39
XP EndoShaper***	10	17.56	9.69	3.46	29.99

^*^Anova p = 0.003

^**^Tukey’s post-hoc test didn’t show significant differences between Reciproc Blue and WaveOne Gold (p = 0.508)

^***^Tukey’s post-hoc test showed significant difference between XP EndoShaper and the other two systems (p < 0.05)

## Discussion

The present controlled randomized study describes and uses a new in vivo method that allows obtaining anatomical measurements, before and after canal preparation, to measure the canal volume increase with 3 different preparation systems: Reciproc Blue (size 25/ 0.08), WaveOne Gold (size 25/ 0.07) and XP Endo Shaper (size 30/ 0.01).

Currently, most of the endodontic NITI systems are designed to provide a round-shaped preparation within oval-shaped canals. Moreover, root canal anatomy goes beyond since it has been reported that root canals have multiple constrictions, pronounced curvatures, and apical foramina with a diameter that oscillates between 0.30 and 0.47 mm [6]. Furthermore, the file tip diameter used in this study is smaller than those of the original anatomy of the root canals, thus generating deficiencies in the debridement of the apical third, which could lead to reduced endodontic therapy success [3]. However, this disadvantage can be compensated due to the reciprocating movement of Reciproc Blue and WaveOne Gold files, which due to the greater contact area between the instrument and the canal walls cut large amounts of dentin [8, 24]. Alternatively, the XP EndoShaper system, because of the novelty of its continuous meandering movement with its booster tip, generates adequate preparations according to the real diameter of the apical foramen and root canal [25].

Currently, several methods such as histological sections, scanning electron microscope, CT, CBCT, and micro CT have been used for reconstructing the anatomy of the original and prepared root canal, in order to evaluate root canal preparation [12]. However, most of these methods are in-vitro and ex-vivo methods such as the Micro-Computed Tomography (micro-CT), which provided significant advances in the three-dimensional reconstruction, with optimal details before, during and after a procedure. Due to its high resolution, it allows to analyze the interior of the evaluated object without damaging it. Unfortunately, it is applicable only to small samples and it cannot be used for in vivo studies in humans [26–28]. Furthermore, in vitro studies may validate techniques and the clinical use of instruments, when they are done under the rigor of the scientific method. These studies help to support clinical models, although their results provide limited analogies to real situations, they give an idea of what can be expected with the clinical use of the instruments [29]. In contrast, this study was aimed to obtain in vivo results, since it presents information in real time with a reproducible method under clinical conditions.

The present study used CBCT as a less-invasive tool, that provides reproducible high resolution and accurate three-dimensional images, allowing to compare the initial root canal morphology with the canal anatomy after preparation [22, 30]. The images obtained were digitized with the Rhinoceros software, for the reconstruction of the root canal through measurements obtained from the tomographic slices [31]. This software provides a practical method to record 3D measurements of study models, its accuracy and reliability allow a realistic and effective measurement, with a margin error of less than 1% [15], constituting it as a reliable and accurate tool for this type of studies.

Measurements were made at different root canal levels, from 0.5, 1, 2, 3, 5 and 7 mm from the root apex, considering that, from 0.5 mm to 3 mm, the anatomical shape of the canal lumen is less oval than the rest, which is an important parameter to consider when analyzing the preparation performed by the different systems. From 5 to 7 mm, the anatomical shape of the canal is more oval [6], representing a challenge to the rotary systems to perform an adequate preparation, due to their tendency to make rounded preparations on the root canal walls [3]. This is an important issue to consider, since this study is in vivo, and therefore subject to anatomical variability.

The initial volume of the pre-instrumented canals was similar for the 3 preparation systems, without showing significant differences between the groups. This analysis is important to verify that the 3 systems worked under similar conditions, in order to guarantee the validity of the results and reducing bias level [24, 32].

Reciproc Blue, presented an average canal volume increase of 110.34%. This could be explained due to its “S” cross-section that has a good cutting ability, its 8% taper at its apical third, and to the reciprocating movement that generates an efficient dentine cutting [33]. WaveOne Gold also showed good cutting ability, although lower than Reciproc Blue, but without showing statistically significant differences, probably due to its 7% taper at its apical third, and its parallelogram cross-section [34]. The XP EndoShaper presented the smallest change in the volume increase (17.55%) of the three preparation systems showing statistically significant differences with respect to the other two instruments. This may be due to the constant 1% taper of the file, together with its high elasticity MaxWire alloy which provokes the file to lengthen and therefore generating less contact on the canal walls [27], making its behavior unpredictable.

To overcome the limitations of this study, future research should be performed on different types of teeth other than premolars with certain degrees of curvature. The control of the double CBCT will always be linked to the comprehensive treatment of the patient, not only to endodontic indications. Up to date, this is the first controlled in vivo clinical trial that was aimed to compare the single-file rotary instrumentation systems under the proposed study model, where their shaping ability was evaluated by measuring canal volume increase, using measurements before and after the in-vivo preparation.

## Conclusion

Within the limitations of this study, it can be concluded that Reciproc-Blue showed higher increase in root canal volume, followed by WaveOne-Gold, while XP-EndoShaper did not significantly increase root canal volume during preparation. The combined use of CBCT and 3D reconstruction with Rhinoceros software, allows the in vivo evaluation of root canal preparation techniques, such as canal volume increase.

## Data Availability

The datasets used and/or analysed during the current study are available from the corresponding author on reasonable request.
